# MOF-Derived Ultrathin Cobalt Phosphide Nanosheets as Efficient Bifunctional Hydrogen Evolution Reaction and Oxygen Evolution Reaction Electrocatalysts

**DOI:** 10.3390/nano8020089

**Published:** 2018-02-07

**Authors:** Hong Li, Fei Ke, Junfa Zhu

**Affiliations:** 1 National Synchrotron Radiation Laboratory and Department of Chemical Physics, University of Science and Technology of China, Hefei 230029, China; hli14@mail.ustc.edu.cn; 2 Department of Applied Chemistry, Anhui Agricultural University, Hefei 230036, China; kefei@ahau.edu.cn

**Keywords:** metal−organic framework, cobalt phosphide, nanosheets, electrocatalyst, hydrogen evolution reaction, oxygen evolution reaction

## Abstract

The development of a highly efficient and stable bifunctional electrocatalyst for water splitting is still a challenging issue in obtaining clean and sustainable chemical fuels. Herein, a novel bifunctional catalyst consisting of 2D transition-metal phosphide nanosheets with abundant reactive sites templated by Co-centered metal−organic framework nanosheets, denoted as CoP-NS/C, has been developed through a facile one-step low-temperature phosphidation process. The as-prepared CoP-NS/C has large specific surface area and ultrathin nanosheets morphology providing rich catalytic active sites. It shows excellent electrocatalytic performances for hydrogen evolution reaction (HER) and oxygen evolution reaction (OER) in acidic and alkaline media, with the Tafel slopes of 59 and 64 mV/dec and a current density of 10 mA/cm^2^ at the overpotentials of 140 and 292 mV, respectively, which are remarkably superior to those of CoP/C, CoP particles, and comparable to those of commercial noble-metal catalysts. In addition, the CoP-NS/C also shows good durability after a long-term test.

## 1. Introduction

Among all the potential clean energy carriers, hydrogen is considered to be ideal since it discharges no harmful chemicals when supplying energy. Therefore, electrochemical reactions, such as the hydrogen evolution reaction (HER) and oxygen evolution reaction (OER) for energy storage systems by electrocatalytic water splitting to generate hydrogen and oxygen, are notable technologies nowadays [1,2,3,4]. Based on a benchmarking protocol, Pt-based materials are ranked as the top catalysts for HER, while RuO_2_ and IrO_2_ are known as the best OER catalysts [5,6]. However, high cost and scarcity of noble metals limit their widespread industrial application. Moreover, separate HER and OER catalysts require different instruments and synthetic approaches, which are not favorable for cost saving. Therefore, the development of high efficient and low-cost bifunctional non-noble-metal electrocatalysts are desirable, and also urgent [7].

Substantial efforts and progress have been devoted to develop non-noble metal-based electrocatalysts for HER and OER with high activity and stability, such as transition metal oxides/sulfides [8,9], metal hydroxides [10], transition metal phosphide [10,11,12], and heteroatom-doped carbon materials [13]. Among them, transition metal phosphide is one of the most intensively investigated bifunctional electrocatalysts for water splitting, such as CoP [14,15], MoP [16], Ni_2_P [17] and FeP [18]. However, the activities of these transition metal phosphides electrocatalysts for water splitting are still much inferior to precious metal-based catalysts, the complicated synthetic procedures and sophisticated fabrication techniques, which further hinder their practical application. Thus, effective and simple fabrication strategies are highly desirable to develop transition metal phosphides electrocatalysts with improved activity and stability.

To achieve high catalytic activity, the catalysts with porous character are essential to expose active sites as much as possible to the electrolyte and substrate. Metal–organic frameworks (MOFs), a family of highly-porous crystalline inorganic−organic hybrid materials with well-defined structures, controllable pore sizes and topologies, and a uniform spatial dispersion of components should be ideal precursors [19]. To use them as highly-efficient electrocatalysts, the material morphology, apart from their composition, needs to be optimized to own a large surface area and porous network architecture for rapid transport of electrons/ions [20,21]. Compared to three-dimensional (3D) nanostructures, two-dimensional (2D) ultrathin nanosheets are particularly attractive for electrocatalysis [22]. This is because ultrathin 2D nanosheets cannot only provide a large area and intimate contact with both the electrolyte and electrode for rapid interfacial charge transfer, but also render more exposed interior atoms as highly-active sites through surface reorganization [23,24]. Recently, greatly enhanced bifunctional catalysts (HER and OER) have been obtained by the pioneering 2D ultrathin nanosheets, such as porous CoP nanosheets [25], FeP nanosheets [18], Ni_2_P nanosheets [26]. Moreover, the existence of carbon in transition metal phosphide complexes may improve the charge transfer efficiency and conductivity in electrocatalysts [27]. Nevertheless, there are only very few reports on carbon-incorporated metallic phosphides nanosheets for bifunctional electrocatalysts.

**Scheme 1:**
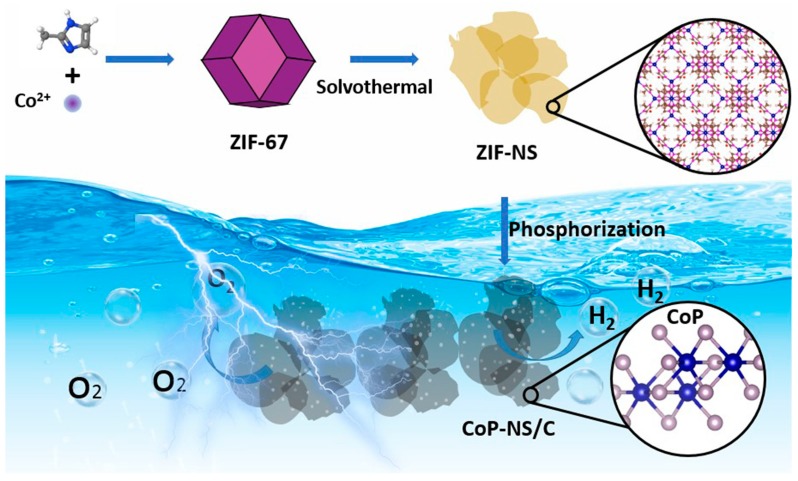
Schematic illustration of the fabrication procedure for CoP-NS/C which used as the HER and OER catalyst.

Herein, we report a one-step synthesis of nanosheets containing cobalt phosphides and amorphous carbon (denoted as CoP-NS/C) through a metal–organic framework (MOF)-based nanosheets strategy. The synthesis process is schematically shown in Scheme 1. In a typical experiment, ZIF-67 were prepared through a reaction of a mixture of Co^2+^ and 2-methyl imidazole. After solvothermal treatment, the ZIF-67 nanosheets (denoted as ZIF-NS) were synthesized, and then the ZIF-NS were transformed into CoP-NS/C through low temperature phosphodation using NaH_2_PO_2_ as the phosphorus source. This is a facile and controllable approach to a new class of metal phosphide urtrathin nanosheets. The MOF-derived porous crystalline CoP nanosheets guarantee highly-exposed active sites, and the existence of carbon contributes to conductive composites, which is crucial for electron transfer. As a result, the optimized CoP-NS/C achieved excellent electrocatalytic performances and high stability for both HER and OER in acidic and alkaline media, respectively. In OER, its electrocatalytic activity was remarkably superior to outperforming commercial IrO_2_.

## 2. Experiment

### 2.1. Materials

All chemicals were commercial and used without further purification: 2-methylimidazole (C_4_H_6_N_2_, 99%), cobalt (II) nitrate hexahydrate (Co(NO_3_)_2_·6H_2_O), methanol (CH_4_O), and sodium hypophosphite monohydrate (Na_2_PO_2_·H_2_O), were purchased from Shanghai Chemical Reagents (Shanghai, China). Commercial Pt/C (20% Pt on Vulcan XC-72), nafion^®^ 117 ~5% in lower aliphatic alcohols and water (Sigma-Aldrich, St. Louis, MI, USA), and potassium hydroxide (KOH, J.T. Baker, Shanghai, China). All other chemicals used in this work were of analytical grade, obtained from commercial suppliers.

### 2.2. Characterizations

The powder X-ray diffraction (PXRD) pattern was collected using a Rigaku SmartLab™ rotation anode X-ray diffractometer (Tokyo, Japan) with graphite monochromatized Cu Kα radiation (1.54178 Å) from 20° to 80°. Transmission electron microscopy (TEM) and high-resolution TEM (HRTEM) were obtained by using a JEOL-2010 TEM (Rigaku, Tokyo, Japan) with an acceleration voltage of 200 kV. Field-emission scanning electron microscopy (FE-SEM) was carried out with a FEI Sirion-200 scanning electron microscope (Rigaku, Tokyo, Japan). Atomic force microscopy (AFM) was carried out on by means of a Veeco DI Nanoscope MultiMode V system (New York, NY, USA). The photoemission spectroscopy experiments were performed at the Catalysis and Surface Science endstation in the National Synchrotron Radiation Laboratory (NSRL), Hefei. The system has been described previously [28]. Briefly, it is equipped with a VG Scienta R4000 analyzer (Uppsala, Sweden), a monochromatic Al Kα X-ray source and a quartz crystal microbalance. The porosity of the sample was measured by nitrogen adsorption/desorption isotherms at 77 K on a Quantachrome autosorb IQ3 (Boynton Beach, FL, USA) and calculated according to the Brunauer-Emmett-Teller (BET) method [29]. Prior to measurement, the samples were degassed overnight at 150 °C under dynamic vacuum which used a Quantachrome masterprep muli-zone flow/vacuum degasser.

### 2.3. Preparation of ZIF-67

The synthesis of ZIF-67 was based on a previous procedure with slight modifications [30]. Typically, 4.32 g of Co(NO_3_)_2_·6H_2_O and 9.75 g of 2-methylimidazole were each dissolved in 300 mL methanol at room temperature. The former solution was poured into the 2-methylimidazole solution with vigorous stirring. The mixture solution was stirred for 0.5 h, and then kept undisturbed at room temperature for 24 h. The violet solid product was obtained by centrifugation and washed with ethanol several times, followed drying at 70 °C for overnight.

### 2.4. Preparation of Co-ZIF Nanosheets (ZIF-NS)

Synthesis of Co-ZIF was similar to the reported method in the literature with some modifications [31]. Co(NO_3_)_3_·6H_2_O (0.546 g) was dissolved in methanol solution (7.5 mL) to form solution A and 2-methyl imidazole (0.616 g) was dissolved in methanol (7.5 mL) to form solution B, solution C was formed by dissolving Co(NO_3_)_3_·6H_2_O (0.546 g) in methanol solution (15 mL). Subsequently, solution A was quickly added into solution B in 1 min, followed by sonication for 5 min. The mixture was washed by methanol using a centrifuge to obtain ZIF-67 particles. Pure ZIF-67 particles were dispersed in the methanol solution (15 mL) and mixed with solution C, under ultrasound conditions for 10 min. The mixture solution was transferred to 50 mL Teflon-lined stainless-steel autoclaves and the reaction was kept at 120 °C for 3 h. Finally, the resultant product was washed three times via centrifugation at 11,000 rpm for 5 min with methanol and, after filtration, the yellow product was put into beakers, followed by subsequent drying at 60 °C in the thermostatic drying oven. The yield of the ZIF-NS sample from ZIF-67 was about 95%.

### 2.5. Synthesis of CoP-NS/C

In a typical synthesis of CoP-NS/C, the obtained ZIF-NS were phosphidated by thermal decomposition of NaH_2_PO_2_ under Ar flow. Twenty milligrams of ZIF-NS and 200 mg NaH_2_PO_2_ were placed at two separate positions in a porcelain boat with NaH_2_PO_2_ at the upstream side of the tube furnace. Then, the samples were annealed at 300 °C for 2 h with a heating rate of 3 °C min^−1^ under an Ar atmosphere. The CoP-NS were obtained after cooling to ambient temperature under Ar. Finally, the impurities and the unstable composition in the sample were washed with water and ethanol twice, respectively. Followed by drying at 60 °C overnight. The yield of the CoP-NS/C sample was about 90%.

### 2.6. Synthesis of CoP

In a typical synthesis of CoP, the obtained CoP-NS were annealed at 250 °C for 3 h in air, and then cooled to room temperature.

### 2.7. Electrochemical Test

#### 2.7.1. Electrochemical Measurements

Electrochemical measurements were performed with a CHI760D electrochemical analyzer (CH Instruments, Inc., Shanghai, China). All electrochemical measurements were conducted in a typical three-electrode setup with a Pt counter electrode and Ag/AgCl reference electrodes. The 0.5 M H_2_SO_4_ solution and 1 M KOH solution were used for the electrochemical measurements. To eliminate the effect of ohmic resistance, all linear sweep voltammetry (LSV) curves are IR corrected for HER and OER data to reflect the intrinsic catalytic behavior. Before the electrochemical activity of the catalyst was recorded, the catalyst was activated by 20 cyclic voltammetry scans at a scan rate of 10 mV/s.

#### 2.7.2. Electrode Preparation

The glassy carbon electrode (GCE, 5 mm in diameter) was polished by powder (1.0 and 0.3 µm alpha alumina, 50 nm gamma alumina, orderly) to obtain a mirror-like surface, then washed with ethanol and distilled water with the assistance of sonication, and blow-dried by N_2_ air flow prior to use, and the surface of the GCE is 0.07 cm^2^. Samples (2 mg) were dissolved in a solvent mixture (1:3, *v*/*v*) of water and ethanol (1 mL) by sonication to form a suspension, then added to 30 µL Nafion solution, and then the solution was sonicated for about 20 min. Subsequently, the catalyst (5 μL) was dropped onto the surface of the pre-polished GCE to form a thin film. Then the electrode was allowed to dry at room temperature for 12 h before measurements (loading was ~0.14 mg/cm^2^).

## 3. Results and Discussion

**Figure 1:**
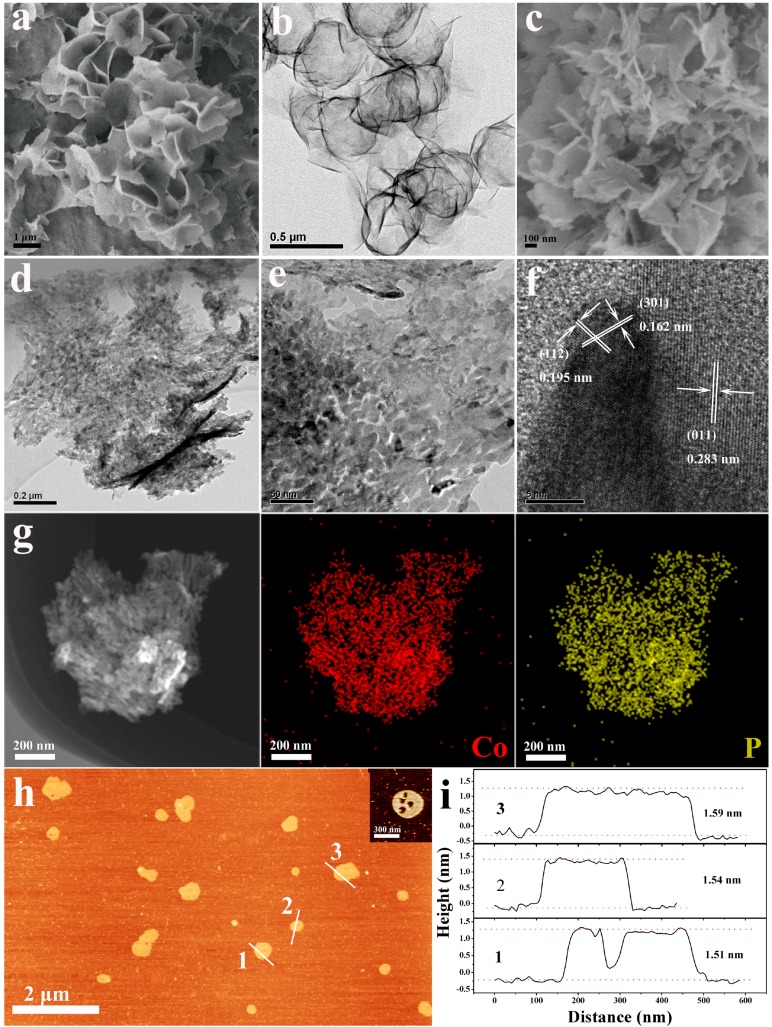
(**a**) Scanning electron microscope (SEM) and (**b**) transmission electron microscopy (TEM) images of ZIF-NS; (**c**) SEM; (**d**,**e**) TEM; and (**f**) high-resolution TEM of the CoP-NS/C; (**g**) the elemental mapping of Co and P elements obtained from the TEM (Co is shown in red, and P in yellow); and (**h**) atomic force microscopy (AFM) images (inset is in high magnification.) of as-prepared CoP-NS/C nanosheets dispersed on a 1 cm × 1 cm mica slice. The corresponding height profiles (**i**) from linescans of selected islands (marked in white line 1, 2 and 3) in Figure 1h.

In a typical experiment, the ZIF-67 regular rhombododecahedron with an average size of 300 nm was firstly synthesized, as confirmed by TEM (Figure S1) and XRD patterns (Figure S2). After 3 h solvothermal treatment, the morphology evolved into numerous ultrathin nanosheets (denoted as ZIF-NS) (Figure 1a,b). The X-ray diffraction (XRD) profiles given in Figure S2 show the appearance of two distinctive diffraction peaks at 20.56° and 33.96° for the newly-developed ZIF-NS, consistent with the previous Yang’s report [31], which confirms the composition and structural evolution. Subsequently, the ZIF-NS were transformed into CoP-NS/C via phosphorization treatment with NaH_2_PO_2_ at 300 °C. Figure 1c,d present typical SEM and TEM images for CoP-NS/C, which shows the morphology of CoP-NS/C is nanosheets. Furthermore, TEM image for an individual CoP-NS/C (Figure 1e) displays a discontinuous and rough surface with some tiny holes, which is quite different from the ZIF-NS with a continuous and smooth surface. The HRTEM image (Figure 1f) exhibits that CoP-NS/C has a lattice fringe with interplanar distances of 0.195, 0.162 and 0.283 nm, corresponding to the (112), (301), and (011) plane of CoP, respectively, which is consistent with the XRD results. The EDX elemental mapping images (Figure 1g) and energy-dispersive X-ray spectroscopy (EDS) spectrum (Figure S3) suggest the presence of Co and P and the uniformly distribution of Co and P elements within the whole nanosheets. The morphology of CoP-NS/C was further examined by atomic force microscopy (AFM), as shown in Figure 1h. The height profiles of the nanoparticles, with respect to the substrate, were analyzed by linescans of three different islands and the results are displayed in Figure 1i. After analyzing the height profiles of all nanosheets in Figure 1h, the average height of the CoP-NS/C is approximately 1.52 ± 0.23 nm, indicating the formation of ultrathin nanosheets. In addition, some holes in the nanosheets can also be observed, as seen in the magnified image in Figure 1h. The formation of in-plane pores is further supported by N_2_ sorption measurements. This implies that the phosphorization treatment of ZIF-NS templates could ‘‘sculpt’’ the sheet to engender more exposed surface area and active sites, which should be beneficial for a high catalytic activity towards the HER and OER.

**Figure 2:**
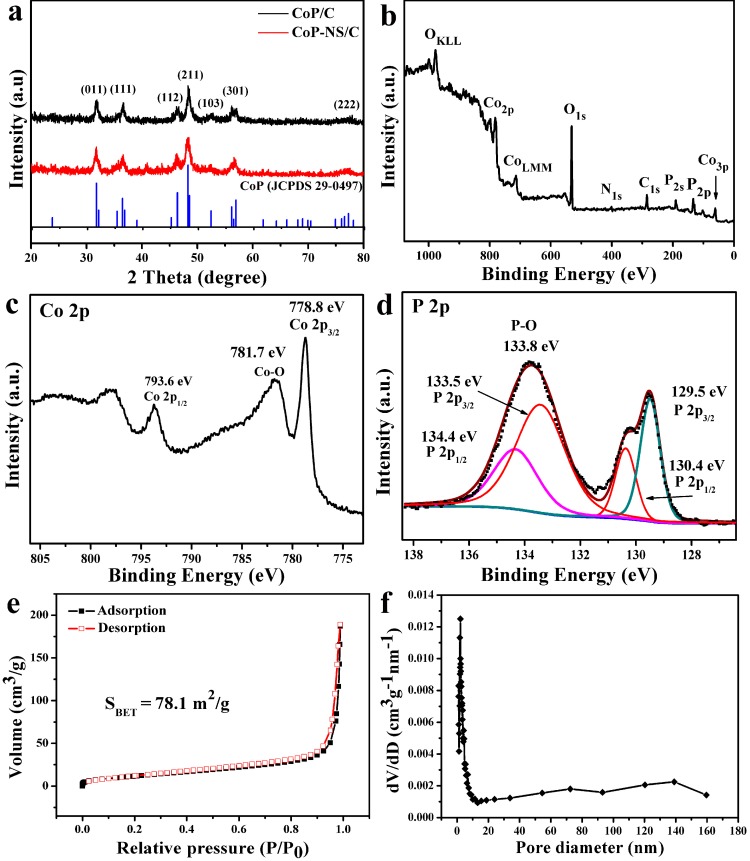
(**a**) X-ray diffraction (XRD) patterns of CoP/C and CoP-NS/C; (**b**) the X-ray photoelectron spectroscopy (XPS) survey spectrum; core-level spectra of (**c**) Co 2p; and (**d**) P 2p; (**e**) N_2_ adsorption/desorption isotherms; and (**f**) BJH pore-size distribution curves of CoP-NS/C. The surface area was calculated using the Brunauer-Emmett-Teller method (SBET = 78.1 m^2^/g). Pore size distributions were calculated using the Barrett-Joyner-Halenda method from the desorption branch. The presence of mesopores with a size of below 20 nm is attributed to pores in the nanosheets. The pore diameter between 20 and 160 nm can be associated with the stacking pores of nanosheets. The pore volume of CoP-NS/C is 0.3 cm^3^/g.

Figure 2a shows the XRD patterns for the CoP-NS/C with the characteristic diffraction peaks at 31.61°, 36.31°, 46.21°, 48.11°, 52.31°, and 56.81° attributable to the (011), (111), (112), (211), (103) and (301) planes, respectively, of CoP (JCPDS-29-0497). Neither a new peak nor a peak shift for the diffraction peaks of CoP was detected, thus suggesting the presence of pure CoP in the CoP-NS/C and CoP/C samples. Further information on the surface composition and valence state of the CoP-NS/C was performed using X-ray photoelectron spectroscopy (XPS). As can be seen in Figure 2b, from XPS survey spectrum, we can observed the existence of Co, P, C, and O elements, among which O element should arise from superficial oxidation of CoP due to air contact, which is in agreement with and EDS spectrum (Figure S3). The Co 2p and P 2p spectra are shown in Figure 2c,d, the existence of Co-O (781.7 eV) and P-O (133.8 eV) bonds agrees with that in survey spectrum as oxidized Co and P species [32]. In addition, Co 2p_3/2_ (778.8 eV) and P 2p_3/2_ (129.5 eV) are close to the binding energy of Co and P in CoP, indicating the formation of CoP in the CoP-NS/C sample [33,34]. The C 1s peaks (Figure S4) at 284.8, 286.4, and 288.8 eV can be assigned to carbon in the form of C–C, C–O, and O=C–O [27,35], respectively, with the C–C peak dominating. Therefore, the existence of carbon in the as-synthesized product after phosphidation of the ZIF-NS is confirmed. There is a significant difference between CoP-NS/C and CoP/C in their specific surface area and porous nature from the N_2_ adsorption/desorption isotherms. CoP-NS/C had a BET surface area of about 78.1 m^2^/g (Figure 2e), contrary to CoP/C particles, only had 25.8 m^2^/g (Figure S6). The Barrett-Joyner-Halenda (BJH) [36] pore-size-distribution curve of CoP-NS/C shows a wide peak ranging from 30 to 160 nm and a narrow peak at about 2 nm with relatively high pore volumes to CoP-NS/C (Figure 2f). Moreover, the above results suggest the holes and cracks on CoP-NS/C would provide large specific surface area and high porosity, thus promoting the transfer of electrons and masses in the electrocatalysis process, which would provide more active sites for HER and OER.

In addition, phosphidation of the ZIF-67 was also carried out at the same conditions (denoted as CoP/C). The presence of the CoP species was proven by the appearance of the characteristic patterns of CoP in the powder XRD profiles (Figure S5a). The TEM image (Figure S5b) indicates that CoP/C has a size of about 100 nm and does not keep the morphology of ZIF-67 precursor. The XPS spectra (Figure S7) suggest that CoP/C shows similar peaks as those of the CoP-NS/C. Moreover, the CoP sample was also synthesized through the calcination of CoP-NS/C in air. The XRD result (Figure S8a) indicates that the CoP has the same crystal structure with CoP (PDF no. 29-0497). The SEM image shown in Figure S8b reveals that CoP keeps the similar nanosheets morphology of CoP-NS/C after air treatment. It is noteworthy that there is no existence of carbon from the XPS survey spectrum (Figure S8c).

**Figure 3:**
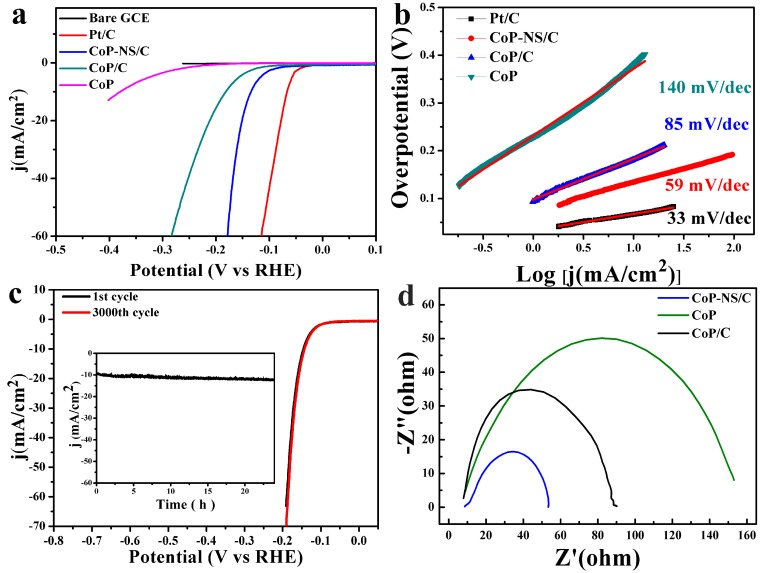
(**a**) IR-corrected polarization curves of CoP-NS/C, CoP/C, CoP, bare GCE (glass carbon electrode) and 20% Pt/C for HER in 0.5 M H_2_SO_4_ at a scan rate of 10 mV/s; (**b**) Tafel slopes of CoP-NS/C, CoP/C, CoP, and 20% Pt/C, respectively; (**c**) polarization curves before and after 3000 CV cycles of the CoP-NS/C ranging from −0.2 to 0 V (inset: time-dependent current density curves under static overpotentials of 140 mV of CoP-NS/C); (**d**) Nyquist plots of the CoP-NS/C, CoP/C and at current density of 10 mA/cm^2^, respectively.

To examine the water splitting performance of obtained catalysts, both cathodic HER and anodic OER processes were evaluated in a three-electrode system with a catalyst loading of 0.14 mg/cm^2^. The electrocatalytic HER activity of the catalysts were tested in 0.5 M H_2_SO_4_ solutions. The results are shown in Figure 3. For comparison, CoP, CoP/C, bare GCE (glass carbon electrode) and commercial Pt/C (20 wt %) were also examined. Figure 3a shows the IR-corrected polarization curves of CoP, CoP/C, CoP-NS/C, bare GCE, and 20% Pt/C. As expected, Pt/C shows the best activity, while a bare GCE shows no catalytic activity. The onset overpotential for CoP-NS/C, CoP/C, CoP, and Pt/C is 77, 101, 219, and 320 mV vs. RHE (reversible hydrogen electrode), in sequence. The CoP-NS/C displays remarkable HER activity, with a low overpotential of 140 mV to achieve 10 mA/cm^2^, much smaller than 188 mV of CoP/C and 383 mV of CoP. This suggests that the HER activity of CoP-NS/C catalyst is superior to those of many other transition metal phosphide (Table S1).The catalysis kinetics for HER can be further examined by Tafel plots (Figure 3b). The linear portions of the Tafel plots were fitted to the Tafel equation (η = *b*log*j + a*, where *j* is the current density, *a* is overpotential when current density is 1 mA/cm^2^ and *b* is the Tafel slope), yielding Tafel slopes of 59, 85, 140 and 33 mV/dec for CoP-NS/C, CoP/C, CoP, and Pt/C, respectively. Furthermore, both CoP-NS/C and CoP/C catalyzed the HER process through the Volmer-Heyrovsky mechanism with an electrochemical desorption process as the rate-determining step. Noticeably, the Tafel slope of CoP-NS/C is much smaller than those of CoP/C and CoP, indicating more favorable kinetics towards the electrocatalytic HER for the CoP-NS/C catalysts. In addition to high HER activity, the stability of CoP-NS/C catalyst also were investigated. Figure 3c depicts the polarization curves before and after 3000 cyclic voltammetry (CV) cycles of the CoP-NS/C catalysts. It can be seen that the polarization curve shows a negligible change compared to the initial curve after 3000 CV sweeps, suggesting the outstanding stability of CoP-NS/C. Moreover, the durability of CoP-NS/C was tested by measuring the time-dependent current density curves under static overpotentials of 140 mV of CoP-NS/C. The result is shown as the inset in Figure 3c. The tiny change of current density over time suggests that CoP-NS/C maintains the excellent catalytic activity for at least 24 h. In addition, the Nyquist plots (Figure 3d) reveal the dramatically-reduced charge transfer resistance (RCT) of the CoP-NS/C relative to the CoP/C and CoP, indicating a much faster electron transfer process and facile kinetics toward hydrogen evolution. Compared to CoP, the existence of carbon in CoP-NS/C and CoP/C may improve the conductivity; on the other hand, compared to CoP/C, 2D nanosheets can provide more active sites for the catalytic process.

**Figure 4:**
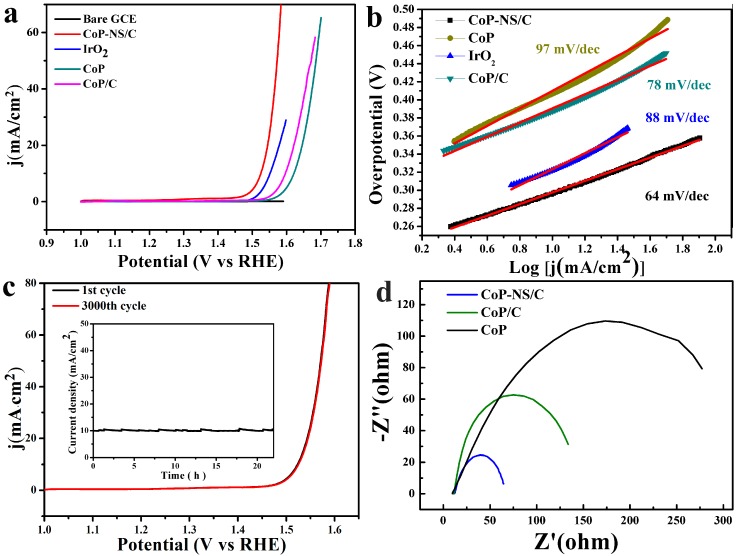
(**a**) IR-corrected polarization curves of CoP-NS/C, CoP/C, CoP, bare GCE, and IrO_2_ for OER in 1 M KOH at a scan rate of 10 mV/s; (**b**) Tafel slopes of CoP-NS/C, CoP/C, CoP, and IrO_2_; (**c**) durability test for the electrochemical OER of CoP-NS/C (inset: time-dependent current density curve of CoP-NS/C under static overpotential of 292 mV); and (**d**) Nyquist plots of the CoP-NS/C, CoP/C and CoP at a current density of 10 mA/cm^2^ in 1 M KOH solution.

Recently, CoP was reported to be a good OER catalyst due to the formation of cobalt oxo/hydroxo species on the catalysts surface [37]. Here, we also investigated the OER catalytic activity of CoP-NS/C together with those of CoP/C, CoP, commercial IrO_2_ and bare GCE electrodes. The results are shown in Figure 4. As expected in Figure 4a, bare GCE has no catalytic activity for OER. The onset overpotentials for CoP-NS/C, IrO_2_, CoP, and CoP/C are 233, 261, 286, and 317 mV (vs. RHE), respectively. The overpotential at current density of 10 mA/cm^2^ for CoP-NS/C is 292 mV, which is lower than those of commercial IrO_2_ (323 mV), CoP/C (382 mV), and CoP (408 mV). Figure 4b are the Tafel plots for CoP-NS/C, IrO_2_, CoP, and CoP/C catalysts. Among all these four catalysts, the Tafel slope of CoP-NS/C (64 mV/dec) is the lowest, as compared to those of IrO_2_ (88 mV/dec), CoP/C (78 mV/dec), and CoP (97 mV/dec), suggesting that the CoP-NS/C catalyst possesses the highest catalytic activity for OER compared with CoP/C, CoP, and IrO_2_. Again, we attributed this property to the unique porous architecture assembled from 2D nanosheets of CoP-NS/C. The durability test for catalyst reveals that although there is no obvious change after 3000 cycles, CoP-NS/C is pretty stable at an overpotential of 292 mV for 24 h and remains at a high activity (Figure 4c). Furthermore, electrochemical impedance spectroscopy (EIS) analysis was performed to study the interfacial properties of catalysts (Figure 4d). Similar to the case of HER, the RCT of CoP-NS/C, indicated by the arc diameter in Nyquist plots, is much smaller than that of CoP/C, suggesting the faster interfacial charge transfer which may be ascribed to the improved conductivity and efficient interfacial contact of 2D porous structure with electrolyte. The OER behavior of CoP-NS/C is comparable and even superior to those of the previously reported CoP nanorods [38], porous CoP polyhedrons [39], CoP hollow polyhedrons [15], and many other transition metal phosphides (Table S2).

According to the above results, we can conclude that the CoP-NS/C catalysts possess the superior electrocatalytic performances as compared to the CoP/C and CoP particles. The origins of the enhanced electrocatalytic activity of CoP-NS/C for HER and OER might be ascribed to the following aspects: (i) the ultrathin nanosheets morphology can facilitate the exposure of more catalytic active sites and transfer of electrons and masses; (ii) the large BET specific surface area and high porosity are expected to improve the charge transfer efficiency; and (iii) the existence of carbon derived from the porous ZIF-NS is expected to improve the charge transfer efficiency and conductivity.

## 4. Conclusions

In summary, cobalt phosphide nanocomposite (CoP-NS/C) fabricated by a novel MOF-templated approach was developed as highly-efficient and durable bifunctional electrocatalysts for both HER and OER. It showed excellent catalytic activity with low overpotentials of 140 and 292 mV to reach a current density of 10 mA/cm^2^ and Tafel slopes of 59 and 64 mV/dec during HER and OER, respectively. The possible origins of these excellent performances are attributed to the synergistic effect, which consists of the MOF derived CoP, the existence of carbon and the porous ultrathin nanosheets structures. In addition, the cost for preparing this material is relatively low and the process is simple, making it promising for practical applications. This study provides a facile strategy for fast synthesis of efficient sheet-like electrocatalysts based on MOF nanosheets. Given the signifiacnt diversity and tailorability of MOFs, the synthesis strategy presented herein holds great promise for the fabrication of new electrocatalysts.

## Supplementary Materials

The following are available online at http://www.mdpi.com/2079-4991/8/2/89/s1; Figure S1: TEM images of ZIF-67; Figure S2: XRD patterns of ZIF-67 simulated, ZIF-67 and ZIF-NS; Figure S3: The energy-dispersive X-ray spectroscopy (EDS) spectrum of CoP-NS/C; Figure S4: High-resolution XPS patterns for C 1s of CoP-NS/C; Figure S5: (a) XRD patterns; and (b) TEM image of CoP/C; Figure S6: N_2_ adsorption/desorption isotherms of CoP/C; Figure S7: XPS spectra of CoP/C: (a) survey spectrum; (b) Co 2p; (c) P 2p; and (d) C 1s; Figure S8: (a) XRD patterns; and (b) SEM image (c) XPS survey spectrum of CoP; Table S1: Comparison of HER performance in 0.5 M H_2_SO_4_ for CoP-NS/C with other transition metal phosphide catalysts; Table S2: Comparison of HER performance in 1 M KOH for CoP-NS/C with other transition metal phosphide catalysts.
