# Supplementary material for: MOF-Derived Ultrathin Cobalt Phosphide Nanosheets as Efficient Bifunctional Hydrogen Evolution Reaction and Oxygen Evolution Reaction Electrocatalysts
Source: Nanomaterials (Basel). 2018 Feb 7;8(2):89. doi: 10.3390/nano8020089 (PMC5853721; doi:10.3390/nano8020089)
Supplement: Supplementary file 1 [file nanomaterials-08-00089-s001.pdf]

nanomaterials

Supporting Information

MOF-Derived Ultrathin Cobalt Phosphide Nanosheets as Efficient Bifunctional Hydrogen Evolution Reaction and Oxygen Evolution Reaction Electrocatalysts

Hong Li [1], Fei Ke [2] and Junfa Zhu [1,*]

[1] National Synchrotron Radiation Laboratory and Department of Chemical Physics, University of Science and Technology of China, Hefei 230029, China; hli14@mail.ustc.edu.cn
[2] Department of Applied Chemistry, Anhui Agricultural University, Hefei 230036, China; kefei@ahau.edu.cn
* Correspondence: jfzhu@ustc.edu.cn; Fax: +86-5-51-5141-078

Supplementary Figures

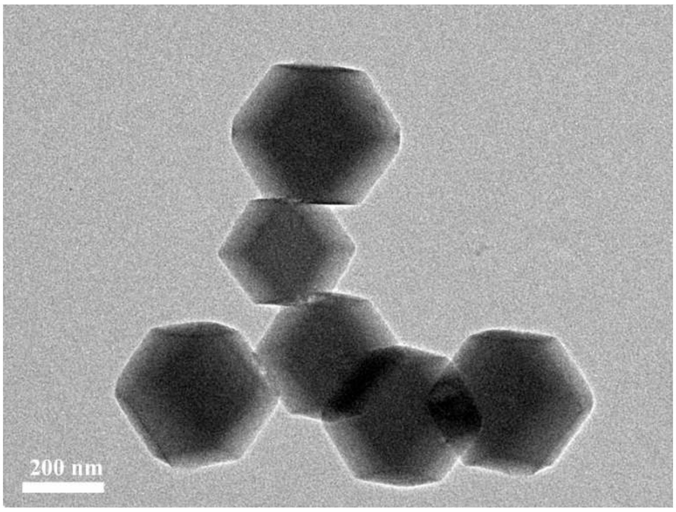

Figure S1. TEM images of ZIF-67.

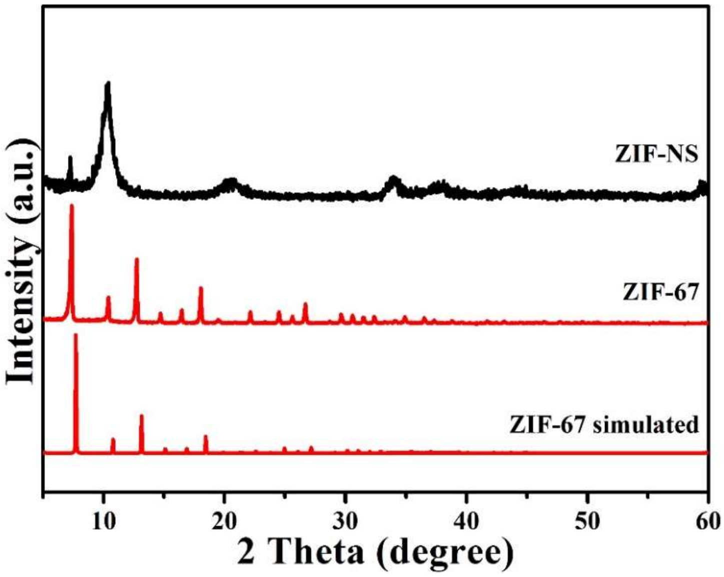

Figure S2. XRD patterns of ZIF-67 simulated, ZIF-67 and ZIF-NS.

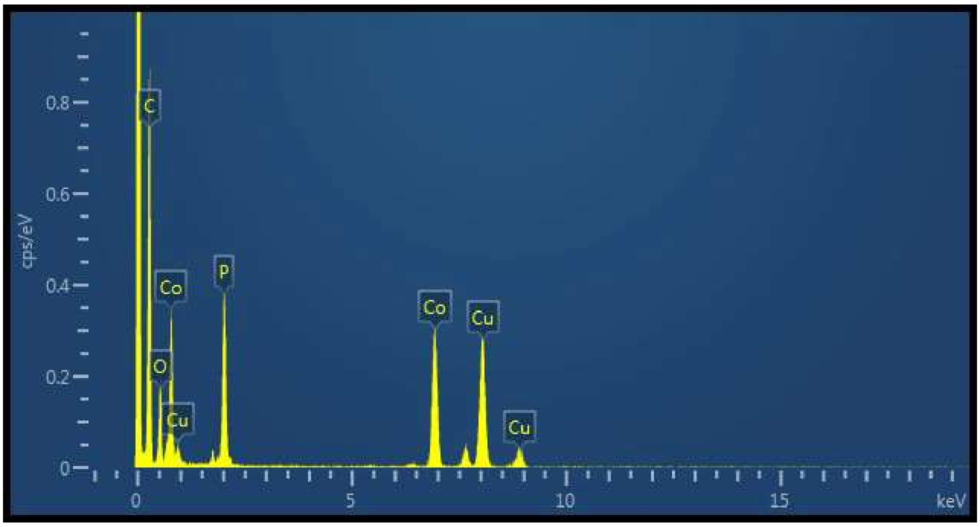

Figure S3. Elements in CoP-NS/C. The energy-dispersive X-ray spectroscopy (EDS) spectrum of CoP-NS/C. EDS spectrum demonstrates the presence of Co and P in as-prepared CoP-NS/C. The signal of Cu results from the copper substrate, the carbon element may come from the carbon tape and sample.

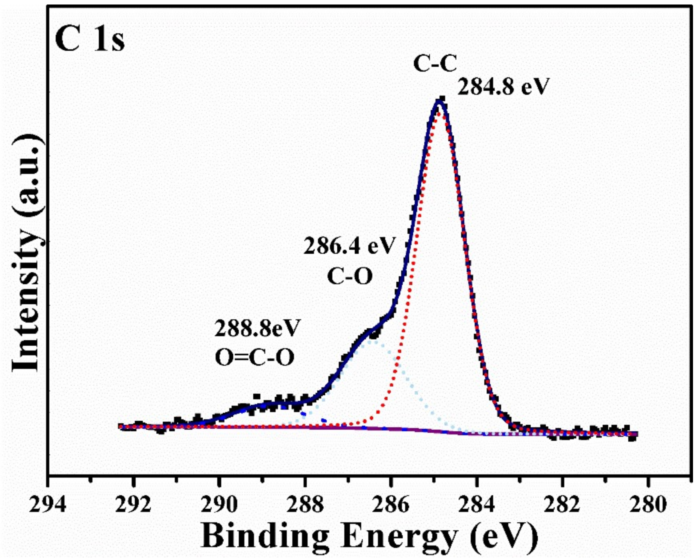

Figure S4. High-resolution XPS patterns for C 1s of CoP-NS/C.

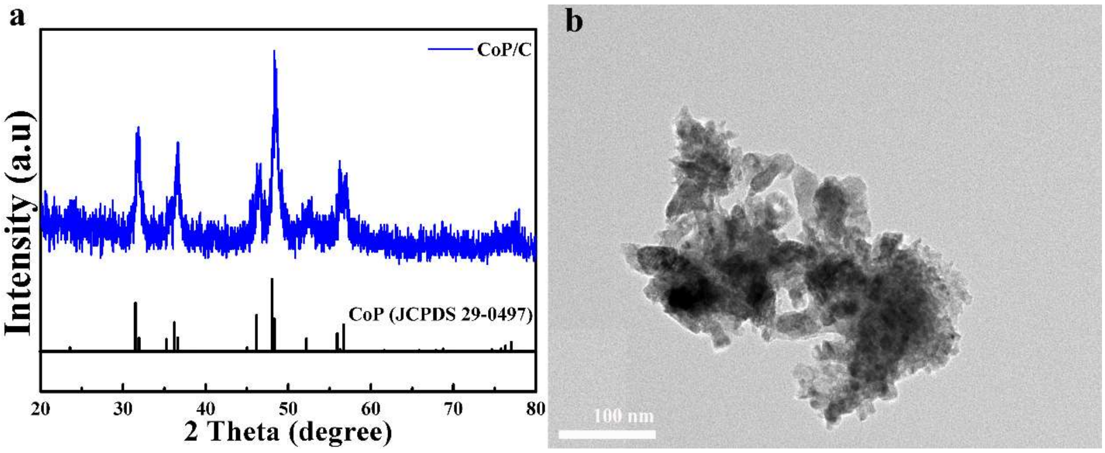

Figure S5. Characterization of CoP/C. (**a**) XRD patterns and (**b**) TEM image of CoP/C. The XRD pattern of the CoP in Figure S4a shows the CoP/C has the same crystal structure with CoP (PDF no.29-0497). The TEM image (Figure S5b) shows that CoP/C had a size of about 100 nm and did not keep the morphology of ZIF-67 precursor.

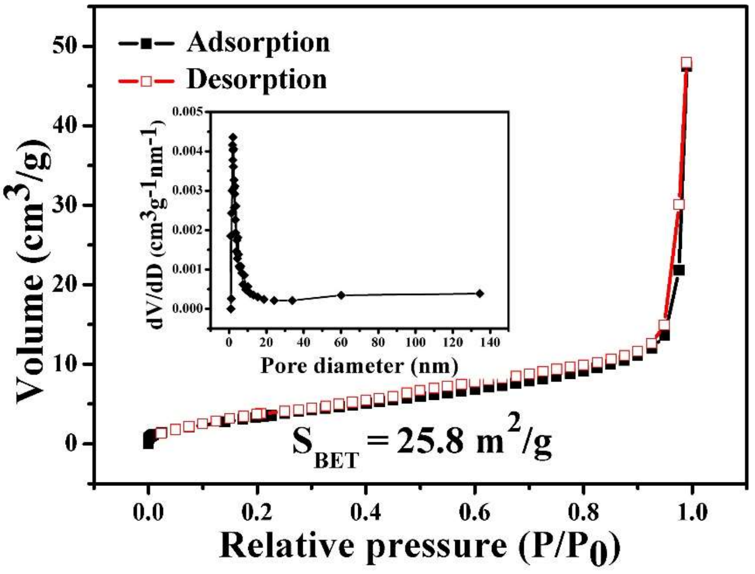

Figure S6. N_2 adsorption/desorption isotherms of CoP/C (inset: BJH pore-size distribution curves). Pore size distributions were calculated using the Barrett-Joyner-Halenda method from the desorption branch. Pore size distribution analysis (Figure S5 inset) reveals a narrow peak of pore diameter distribution ranging from 0.9 to 10 nm. The Brunauer–Emmett–Teller (BET) surface area (S_{BET}) and pore volume of the CoP/C were calculated to be 25.8 m^2/g and 0.08 cm ^{3}/g, respectively.

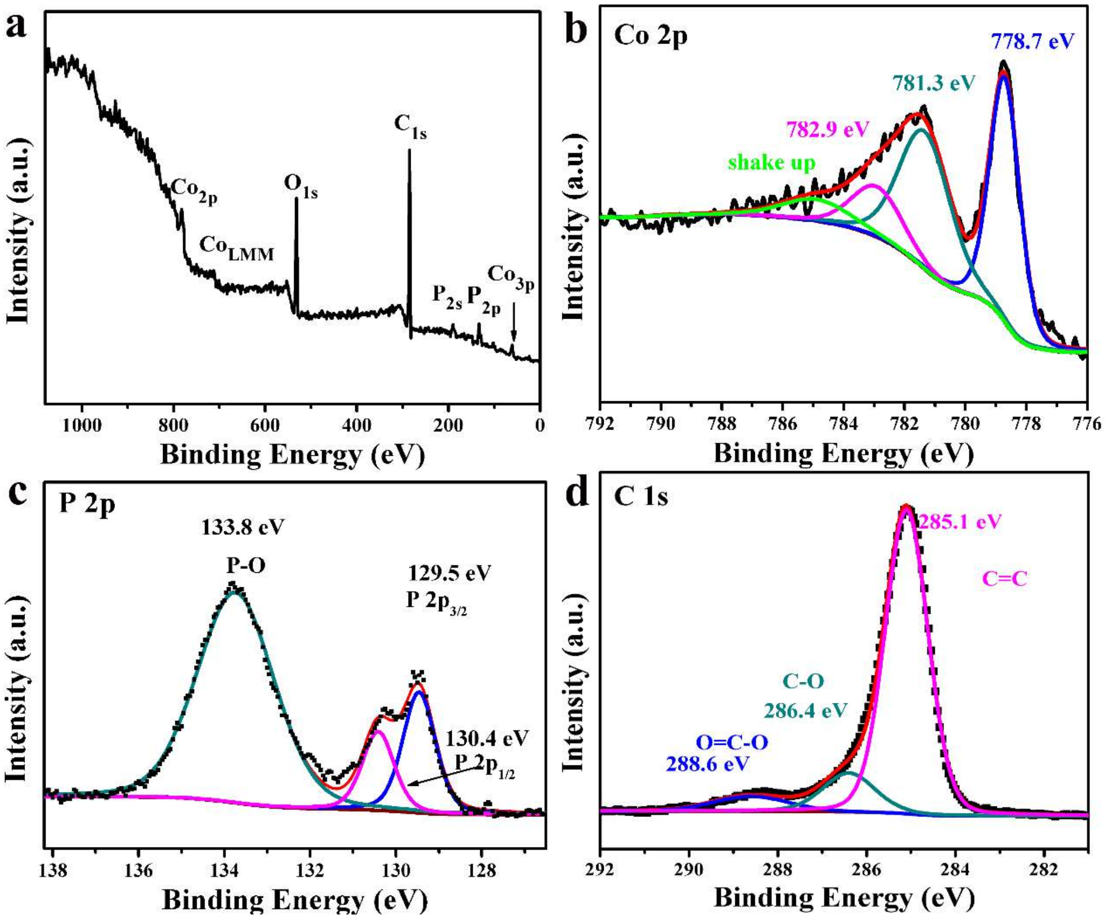

Figure S7. XPS spectra of CoP/C: (**a**) survey spectrum; (**b**) Co 2p; (c) P 2p and (d) C 1s.

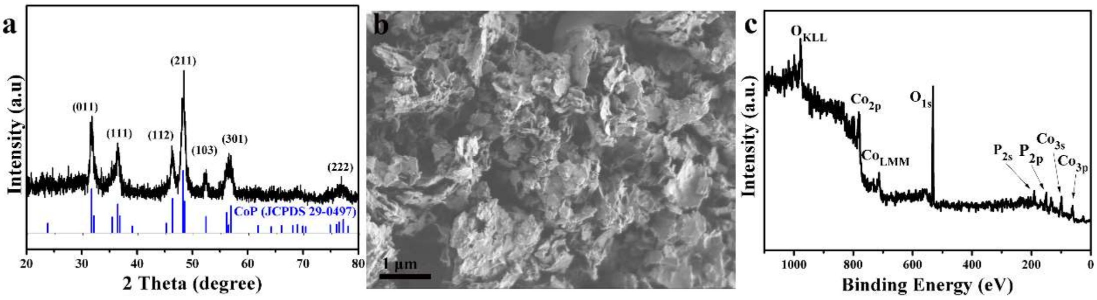

Figure S8. (**a**) XRD patterns and (**b**) SEM image and (**c**) XPS survey spectrum of CoP. The XRD pattern of CoP in (a) shows that the as-prepared CoP has the same crystal structure with CoP (PDF no.29-0497) and no other peak were observed. XPS survey spectrum of CoP confirmed no existence of carbon in CoP.

Table S1. Comparison of HER performance in 0.5M H_2SO_4 for CoP-NS/C with other transition metal phosphide catalysts.

Catalyst for HER	Catalyst Size (nm)	Overpotential for 10 mA/cm^2 (mV)	Tafe slope (mV/dec)	Catalyst loading (mg/cm^2)	Refs.
CoP Hollow Polyhedron	700	206	39	0.102	[1]
CoP nanosheet	1.1 (thickness)	56	44	0.28	[2]
Co phosphide/Co phosphate	80	160	53	-	[3]
urchin-like CoP	4000	100	46	0.28	[4]
Ni_5P_4-Ni_2P-NS array	-	120	79.1	68.2	[5]
CoP@C	20–50	170	61	0.353	[6]
CoP/RGO	4.1	250	104.8	0.29	[7]
Co_2P nanorods	110.0 ± 11.8 (length) 9.8 ± 1.3 (diameter)	134	71	1	[8]
$Ni_{12}P_5$/Ti	14.3 ± 2.0	137	63	1	[9]
Ni_2P nanosheets/CC	-	99	51	4.3	[10]
Cu_3P NW/CF	Several micrometers (length) 300–400 (diameter)	143	67	15.2	[11]
MoP@PC	-	153	66	0.41	[12]
FeP nanosheets	-	220	67	0.28	[13]
CoP-NS/C	1.52 ± 0.23 (thickness)	140	59	0.14	This work

Table S2. Comparison of OER performance in 1M KOH for CoP-NS/C with other transition metal phosphide catalysts.

**Catalyst for OER**	**Catalyst Size (nm)**	**Overpotential for 10 mA/cm^2 (mV)**	**Tafe slope (mV/ dec)**	**Catalyst loading (mg/cm^2)**	**Refs.**
Co-P film	1000–3000	345	47	-	[14]
Co phosphide/Co phosphate	80	310	65	-	[3]
$Cu_{0.3}Co_{2.7}P$/NC	500	190	44	0.4	[15]
$Ni_{0.69}Co_{0.31}$–P	less than 10	266	81	3.5	[16]
NiCoP nanosheet arrays	6000–8000	308(50)	-	5	[17]

$(Ni_{0.5}Fe_{0.5})_2P$/Ni foam	-	203	57	-	[18]
$Fe_{10}Co_{40}Ni_{40}P$/Ni foam	-	250	44	3.1	[19]
CoP nanorod	-	320	71	0.71	[20]
Co-P/NC	600	319	52	0.283	[21]
NiCoP/C nanoboxes	750	330	96	-	[22]
CoP/RGO	200	340	70	0.29	[23]
CoP Hollow Polyhedron	700	400	57	0.102	[1]
CuP microsheets	510	290	63	-	[24]
Ni_2P nanosheets	-	347	63	0.285	[25]
FeP @CNT	-	300	53	0.204	[26]
CoP-NS/C	1.52 ± 0.239 (thickness)	292	64	0.14	This work

References

1. Liu, M.; Li, J. Cobalt phosphide hollow polyhedron as efficient bifunctional electrocatalysts for the evolution reaction of hydrogen and oxygen. *Acs Appl. Mater. Inter.* **2016**, *8*, 2158–2165.
2. Zhang, C.; Huang, Y.; Yu, Y.; Zhang, J.; Zhuo, S.; Zhang, B. Sub-1.1 nm ultrathin porous CoP nanosheets with dominant reactive {200} facets: A high mass activity and efficient electrocatalyst for the hydrogen evolution reaction. *Chem. Sci.* **2017**, *8*, 2769–2775.
3. Yang, Y.; Fei, H.; Ruan, G.; Tour, J.M. Porous cobalt-based thin film as a bifunctional catalyst for hydrogen generation and oxygen generation. *Adv. Mater.* **2015**, *27*, 3175–3180.
4. Yang, H.; Zhang, Y.; Hu, F.; Wang, Q. Urchin-like CoP nanocrystals as hydrogen evolution reaction and oxygen reduction reaction dual-electrocatalyst with superior stability. *Nano Lett.* **2015**, *15*, 7616–7620.
5. Wang, X.; Kolen'ko, Y.V.; Bao, X.Q.; Kovnir, K.; Liu, L. One-step synthesis of self-supported nickel phosphide nanosheet array cathodes for efficient electrocatalytic hydrogen generation. *Angew. Chemie Int. Ed.* **2015**, *54*, 8188–8192.
6. Wang, C.; Jiang, J.; Zhou, X.; Wang, W.; Zuo, J.; Yang, Q. Alternative synthesis of cobalt monophosphide@ C core–shell nanocables for electrochemical hydrogen production. *J. Power Sources* **2015**, *286*, 464–469.
7. Ma, L.; Shen, X.; Zhou, H.; Zhu, G.; Ji, Z.; Chen, K. CoP nanoparticles deposited on reduced graphene oxide sheets as an active electrocatalyst for the hydrogen evolution reaction. *J. Mater. Chem. A* **2015**, *3*, 5337–5343.
8. Huang, Z.; Chen, Z.; Chen, Z.; Lv, C.; Humphrey, M.G.; Zhang, C. Cobalt phosphide nanorods as an efficient electrocatalyst for the hydrogen evolution reaction. *Nano Energy* **2014**, *9*, 373–382.
9. Huang, Z.; Chen, Z.; Chen, Z.; Lv, C.; Meng, H.; Zhang, C. $Ni_{12}P_5$ nanoparticles as an efficient catalyst for hydrogen generation via electrolysis and photoelectrolysis. *ACS Nano* **2014**, *8*, 8121–8129.
10. Jiang, P.; Liu, Q.; Sun, X. NiP_2 nanosheet arrays supported on carbon cloth: An efficient 3d hydrogen evolution cathode in both acidic and alkaline solutions. *Nanoscale* **2014**, *6*, 13440–13445.
11. Tian, J.; Liu, Q.; Cheng, N.; Asiri, A.M.; Sun, X. Self-supported Cu_3P nanowire arrays as an integrated high-performance three-dimensional cathode for generating hydrogen from water. *Angew. Chemie Int. Ed.* **2014**, *53*, 9577–9581.
12. Yang, J.; Zhang, F.; Wang, X.; He, D.; Wu, G.; Yang, Q.; Hong, X.; Wu, Y.; Li, Y. Porous molybdenum phosphide nano-octahedrons derived from confined phosphorization in UIO-66 for efficient hydrogen evolution. *Angew. Chemie* **2016**, *128*, 13046–13050.
13. Xu, Y.; Wu, R.; Zhang, J.; Shi, Y.; Zhang, B. Anion-exchange synthesis of nanoporous FeP nanosheets as electrocatalysts for hydrogen evolution reaction. *Chem. Commun.* **2013**, *49*, 6656–6658.
14. Jiang, N.; You, B.; Sheng, M.; Sun, Y. Electrodeposited cobalt-phosphorous-derived films as competent bifunctional catalysts for overall water splitting. *Angew. Chemie* **2015**, *127*, 6349–6352.
15. Song, J.H.; Zhu, C.Z.; Xu, B.Z.; Fu, S.F.; Engelhard, M.H.; Ye, R.F.; Du, D.; Beckman, S.P.; Lin, Y.H. Bimetallic cobalt-based phosphide zeolitic imidazolate framework: CoP_x phase-dependent electrical conductivity and hydrogen atom adsorption energy for efficient overall water splitting. *Adv. Energy Mater.* **2017**, *7*.
16. Yin, Z.; Zhu, C.; Li, C.; Zhang, S.; Zhang, X.; Chen, Y. Hierarchical nickel–cobalt phosphide yolk–shell spheres as highly active and stable bifunctional electrocatalysts for overall water splitting. *Nanoscale* **2016**, *8*, 19129–19138.
17. Yu, J.; Cheng, G.; Luo, W. Hierarchical NiFeP microflowers directly grown on Ni foam for efficient electrocatalytic oxygen evolution. *J. Mater. Chem. A* **2017**, *5*, 11229–11235.

18. Li, Y.; Zhang, H.; Jiang, M.; Kuang, Y.; Sun, X.; Duan, X. Ternary NiCoP nanosheet arrays: An excellent bifunctional catalyst for alkaline overall water splitting. *Nano Res.* **2016**, *9*, 2251–2259.
19. Zhang, Z.; Hao, J.; Yang, W.; Tang, J. Iron triad (Fe, Co, Ni) trinary phosphide nanosheet arrays as high-performance bifunctional electrodes for full water splitting in basic and neutral conditions. *RSC Adv.* **2016**, *6*, 9647–9655.
20. Chang, J.; Xiao, Y.; Xiao, M.; Ge, J.; Liu, C.; Xing, W. Surface oxidized cobalt-phosphide nanorods as an advanced oxygen evolution catalyst in alkaline solution. *ACS Catal.* **2015**, *5*, 6874–6878.
21. You, B.; Jiang, N.; Sheng, M.; Gul, S.; Yano, J.; Sun, Y. High-performance overall water splitting electrocatalysts derived from cobalt-based metal–organic frameworks. *Chem. Mater.* **2015**, *27*, 7636–7642.
22. He, P.; Yu, X.Y.; Lou, X.W. Carbon-incorporated nickel-cobalt mixed metal phosphide nanoboxes with enhanced electrocatalytic activity for oxygen evolution. *Angew. Chem. Int. Ed.* **2017**, *56*, 3897–3900.
23. Jiao, L.; Zhou, Y.-X.; Jiang, H.-L. Metal–organic framework-based CoP/reduced graphene oxide: High-performance bifunctional electrocatalyst for overall water splitting. *Chem. Sci.* **2016**, *7*, 1690–1695.
24. Hao, J.; Yang, W.; Huang, Z.; Zhang, C. Superhydrophilic and superaerophobic copper phosphide microsheets for efficient electrocatalytic hydrogen and oxygen evolution. *Adv. Mater. Interfaces* **2016**, *3*.
25. Li, Z.; Dou, X.; Zhao, Y.; Wu, C. Enhanced oxygen evolution reaction of metallic nickel phosphide nanosheets by surface modification. *Inorg. Chem. Front.* **2016**, *3*, 1021–1027.
26. Yan, Y.; Zhao, B.; Yi, S.C.; Wang, X. Assembling pore-rich FeP nanorods on the CNT backbone as an advanced electrocatalyst for oxygen evolution. *J. Mater. Chem. A* **2016**, *4*, 13005–13010.2.
